# Data on Gabonese rodents and their Plasmodium

**DOI:** 10.1016/j.dib.2019.104646

**Published:** 2019-10-15

**Authors:** Larson Boundenga, Diamella-Nancy Moukodoum, Barthélémy Ngoubangoye

**Affiliations:** Centre International de Recherches Médicales de Franceville (CIRMF), BP: 769 Franceville, Gabon

**Keywords:** Molecular tools, Plasmodium, Rodents, Gabon, Cytochrome-b and tissues

## Abstract

In this paper present data on the description of rodent species living around human dwelling in some villages of Gabon and their malaria parasites. Rodents are known to colonize various environments, such as forest; domestic or peridomestic environment. They are known to be the hosts of many parasites. Data presented here the circulation of malaria parasites in Gabonese rodents was shown; the estimation of pairwise genetic distances (*p*-distance) between rodents malaria parasites. We also provide data on rodent species diversity in Gabon. Three hundred and forty-five samples from rodents conserved in biobank of International Center of Medical Researches of Franceville (CIRMF) were used for the study. These samples were collected in six villages of southeastern of Gabon between 2009 and 2016 for routine monitoring of infectious disease. Such data can be used to describe and understanding the evolution and systematics of malaria parasite. This data set support the main findings presented in the research article [1].

**Table:** Specifications Table

Subject	Ecology, Evolution, Behaviour and Systematics
Specific subject area	Molecular tools, Parasitology, Phylogenetic analyses, Ecology
Type of data	Table Image Figure
How data were acquired	All samples were collected in different province of Gabon, molecular tools, PCR, sequencing (sanger method) and phylogenetic analyzes (PhyML and Bioedit)
Data format	Raw and Analyzed
Parameters for data collection	DNA was extracted using whole blood or Tissue (liver/spleen); morphometric data,
Description of data collection	portion of mitochondrial gene cytochrome b
Data source location	International Center for the Medicals Research of Franceville (CIRMF),Franceville/Haut-Ogooue Gabon
Data accessibility	The data is available within this article and NCBI Genbank: MK395253 to MK395265 for Plasmodium and MK519268 to MK519280 for rodents.
Related research article	L. Boundenga, B. Ngoubangoye, S. Ntie, N. Moukodoum, F. Renaud, V. Rougeron, F. Prugnolle. Rodent malaria in Gabon: diversity and host range, Int J Parasitol Parasites Wildl., 10, 2019, 117–124 [1].

**Table:** 

**Value of the Data** • This data is important for the researchers who work or would like to work in field of evolution of malaria parasites or on ecology of rodents. • These data provide several sequences of malaria parasites found in rodents living in peri domestic areas throughout Gabon and could be used in further investigation as base • Here we report the cytochrome-b sequences for five central African rodent species, as well as morphometric data for each species. • The diagnostic method developed here in this study could be useful towards other investigations on parasites circulating in blood.

## 1 Data

**Fig. 1:**
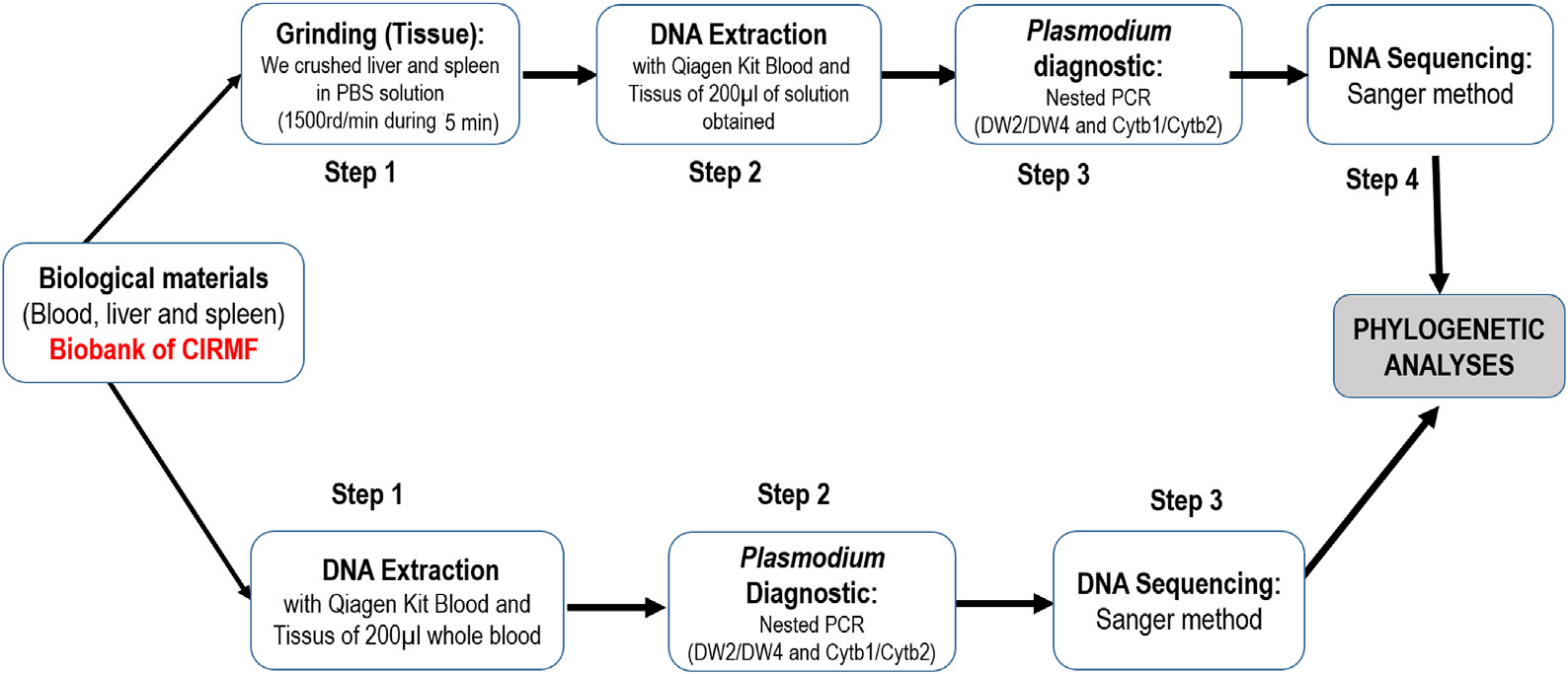
Illustration of the different steps of *Plasmodium* diagnostic in mammals used whole blood or organs (liver/spleen) for DNA extraction. This methods was more explained in our previous studies [2,3] where we used firstly this protocol to identification of malaria parasites in wildlife.

**Fig. 2:**
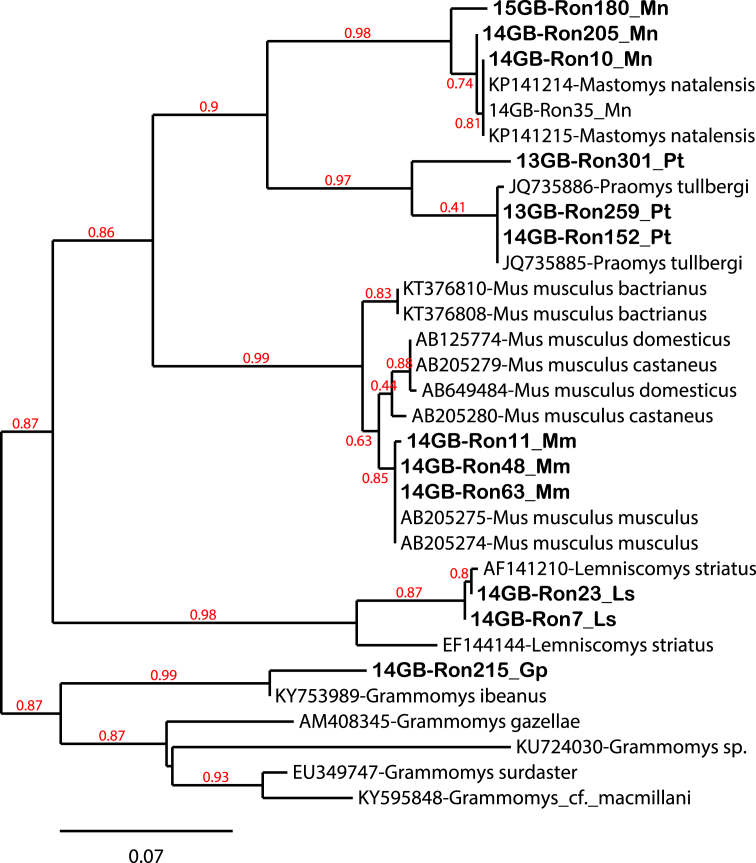
Phylogenetic relationships between the Cyt-b sequences of rodents obtained in our study (bold) and the others sequences from existing databases. The tree was built based on partial sequences of Cyt-b (750 bp-long) using PhyML (freely available at the ATGC bioinformatics platform http://www.atgc-montpellier.fr/) using NNI (Nearest Neighbor Interchange) + SPR (Subtree Pruning Regrafting) branch swapping and 100 bootstrap replicates. The names of our isolates (for instance, n14GB-Ron48_Mus musculus-DJM) include: 1) the year and country of collection (n14GB: n14: 2014 and GB: Gabon); 2) the sample number (Ron48: Rodent number 48); 3) the rodent species and 4) the abbreviation of the sample site (FCV: Franceville; MIM: Mimongo, LEK: Lekoni, DJM: Djoumou; MKK: Makokou; KLM: Koulamoutou).

The dataset presented here describes methods of identification of the rodent diversity and Plasmodium species infecting the rodents dwelling peri-domestic environment. Fig. 1 describes different steps of characterization of malaria parasites using whole blood or organs (liver/spleen). Fig. 2 described phylogenetic relationships between the rodents captured in Gabon and other from other countries from Genbank using a portion of mitochondrial gene (Cyt-b). Table 1 describes the diversity and percentage each parasite obtained according to the material used and the infected host species. Table 2 presents results of molecular characterization of the species of the rodents. Table 3 presents results of estimation of the pairwise genetic distance (*p*-distances) between cytochrome b of Plasmodium lineages obtained and others lineages indexed in Genbank and Table 4 S1 presents complete data base of captured rodents.

**Table 1:** Describes of the diversity and percentage of *Plasmodium* species identified.

*Plasmodium* species	Biological materials				
	Whole blood (N = 60)	Liver and spleen (N = 285)	Rodent species infected	Percentage (%)	Accession number of the *Plasmodium* detected
*Plasmodium vinckei*	3/60	5/285	- *Mastomy natalensis*	2.31 (8/345)	*MK519275; MK519276; MK519274; MK519273*
			- *Mus musculus*		*MK519280*
			- *Praomys* sp.		*MK519271*
			- *Lemniscomys striatus*		*MK519268*
			- *Grammomys poensis*		*MK519277*
*Plasmodium yoelii*	1/6	2/285	- *Praomys* sp.	0.86 (3/345)	*MK519270; MK519272*
			- *Lemniscomys striatus*		*MK519269*
*P.* sp. GAB	1/285	1/285	- *Mus musculus*	0.57 (2/345)	*MK519279; MK519278*

**Table 2:** Results of molecular characterization of the species of the rodents. This table consider inly the positive individual of our study. The species were identified using the methods described in [5,6]. Thus our data show the presence of these species in the peri-domestic environment of Gabon.

Identifier	Year of collection	Localization	Species identification	Genbank number	Gene analyzed
14GB-Ron7	2014	Franceville	*Lemniscomys striatus*	MK519268	Cytochrome b
14GB-Ron23	2014	Franceville	*Lemniscomys striatus*	MK519269	Cytochrome b
13GB-Ron301	2013	Franceville	*Proamys* sp.	MK519270	Cytochrome b
13GB-Ron259	2013	Lekoni	*Proamys* sp.	MK519271	Cytochrome b
14GB-Ron152	2011	Lekoni	*Proamys* sp.	MK519272	Cytochrome b
14GB-Ron10	2014	Koulamoutou	*Mastomys natalnsis*	MK519273	Cytochrome b
14GB-Ron35	2013	Lekoni	*Mastomys natalnsis*	MK519274	Cytochrome b
15GB-Ron180	2015	Makokou	*Mastomys natalnsis*	MK519275	Cytochrome b
14GB-Ron205	2014	Makokou	*Mastomys natalnsis*	MK519276	Cytochrome b
14GB-Ron215	2014	Makokou	*Grammomys poensis*	MK519277	Cytochrome b
14GB-Ron11	2014	Djoumou	*Mus musculus*	MK519278	Cytochrome b
14GB-Ron63	2014	Mimongo	*Mus musculus*	MK519279	Cytochrome b
14GB-Ron48	2014	Djoumou	*Mus musculus*	MK519280	Cytochrome b

**Table 3:** The pairwise genetic distance (*p*-distances) between cytochrome b of *Plasmodium* lineages obtained in rodents samples shown in Table 1. This estimation was made using Kimura two-parameter model of substitutions.

Parasite species	1	2	3	4	5	6	7	8	9	10	11	12	13	14	5	16	17	18	19	20	21	22	23
(1) 14GB-Ron152_M_muscullus																							
(2) 14GB-Ron7_L_striatus	0,06																						
(3) 14GB-Ron63_M_muscullus	0,03	0,08																					
(4) 14GB-Ron11_M_muscullus	0,03	0,08	0,00																				
(5) 14GB-Ron23_L_striatus	0,00	0,06	0,03	0,03																			
(6) 14GB-Ron48_M_muscullus	0,06	0,01	0,08	0,08	0,07																		
(7) 14GB-Ron10_M_natalensis	0,06	0,00	0,08	0,08	0,06	0,01																	
(8) 14GB-Ron35_M_natalensis	0,06	0,00	0,08	0,08	0,06	0,01	0,00																
(9) 15GB-Ron180_M_natalensis	0,06	0,00	0,07	0,07	0,06	0,01	0,00	0,00															
(10) 14GB-Ron205_M_natalensis	0,06	0,00	0,08	0,08	0,06	0,01	0,00	0,00	0,01														
(11) DQ414654-P. v._lentum	0,06	0,01	0,08	0,08	0,07	0,02	0,01	0,01	0,01	0,01													
(12) DQ414653-P. v._lentum	0,06	0,01	0,08	0,08	0,07	0,02	0,01	0,01	0,01	0,01	0,00												
(13) DQ414655-P. v._petteri	0,06	0,03	0,08	0,08	0,06	0,04	0,03	0,03	0,03	0,03	0,04	0,04											
(14) DQ414656-P. v._petteri	0,06	0,03	0,08	0,08	0,06	0,04	0,03	0,03	0,03	0,03	0,04	0,04	0,00										
(15) DQ414650-P. vinckei	0,06	0,03	0,08	0,08	0,06	0,04	0,03	0,03	0,03	0,03	0,04	0,04	0,00	0,00									
(16) DQ414652-P. vinckei	0,06	0,05	0,08	0,08	0,06	0,05	0,05	0,05	0,05	0,05	0,05	0,05	0,04	0,04	0,04								
(17) DQ414651-P. v._vinckei	0,06	0,05	0,07	0,07	0,07	0,06	0,05	0,05	0,05	0,05	0,05	0,05	0,05	0,05	0,05	0,05							
(18) DQ414659-P. y_nigeriensis	0,00	0,06	0,03	0,03	0,00	0,07	0,06	0,06	0,06	0,06	0,07	0,07	0,06	0,06	0,06	0,06	0,07						
(19) DQ414660-P. y._yoelii	0,00	0,06	0,03	0,03	0,00	0,07	0,06	0,06	0,06	0,06	0,07	0,07	0,06	0,06	0,06	0,06	0,07	0,00					
(20) AY099051-P. yoelii	0,00	0,06	0,03	0,03	0,00	0,07	0,06	0,06	0,06	0,06	0,07	0,07	0,06	0,06	0,06	0,06	0,07	0,00	0,00				
(21) DQ414658-P. y._killicki	0,01	0,06	0,03	0,03	0,01	0,07	0,06	0,06	0,06	0,06	0,07	0,07	0,06	0,06	0,06	0,06	0,06	0,01	0,01	0,01			
(22) DQ414657-P. yoelii-EL	0,00	0,06	0,03	0,03	0,00	0,07	0,06	0,06	0,06	0,06	0,07	0,07	0,06	0,06	0,06	0,06	0,07	0,00	0,00	0,00	0,01		
(23) AY099054-P. atheruri	0,06	0,03	0,08	0,08	0,06	0,04	0,03	0,03	0,03	0,03	0,04	0,04	0,00	0,00	0,00	0,04	0,05	0,06	0,06	0,06	0,06	0,06	

## 2 Experimental design, materials, and methods

All rodents were captured using Tomahawk and Shermann traps in peri-domestic habitats (up to 250 m from the houses). The traps being set inside and around human dwellings, in each city. For each individual, species or genus identification of the rodent was based on morphological characters and the parameters like sex, age, weight or morphometric limbs (foot and arm) were taken (Table S1). After the euthanasia, samples of different organs were collected (liver, spleen, kidney, lung, heart, intestine and brain), frozen and transported to the Centre International de Recherches Médicales de Franceville. Finally, the collected samples were stored at −80 °C until needed for molecular analyses.

Total DNA, for each selected animal was extracted from approximately 100 mg of organ tissue (spleen or liver) mixed with 500 μl of PBS solution or 200μl of blood according to the procedures described by Boundenga et al. [2,3]. We ground the samples on an automaton set at 2000 rpm for 5 minutes, then we used 200 μl from each sample for DNA extraction (Fig. 1). Total DNA was extracted from with the DNeasy Blood and Tissue Kit (Qiagen, Courtaboeuf, France) and used as template for the detection of Plasmodium parasites of rodents according to a previously described protocol [3]. For amplification of malaria parasites, a nested PCR was performed on each sample targeting a ∼930bp fragment of the Plasmodium cytochrome b (cyt-b) gene is based on a nested PCR with 2 sets of primers such as described in Ref. [4].

The first was developed by Perkins and Schall (2002) (DW2 5′-TAATGCCTAGACGTATTCCTGATTATCCAG-3′ and DW4 5′-TGTTTGCTTGGGAGCTGTAATCATAATGTG-3′). For this first round, we used 2 μl of DNA template in a 20 μl reaction volume, containing: 4 μl of 5 × Reaction Buffer, 1.5 mM MgCl2, 200 μM of each dNTP, 20 pmol of each primer (DW2 and DW4), and 2.5 U Taq DNA Polymerase (Promega). Cycling conditions for the first round were as follows: 3 min at 94 °C; 20 sec at 94 °C; 20 sec at 60 °C; 1 min 30 sec at 72 °C (repeated for 35 cycles); 10 min at 72 °C. For the second round of Cyt b gene amplification, we used the primers from Schwöbel et al. (2003) (Cytb1 5′-CTCTATTAATTTAGTTAAAGCACA-3′ and Cytb2 5′-ACAGAATAATCTCTAGCACC-3′) and we used 1 μl of 1st PCR template in a 25 μl reaction volume, containing: 5 μl of 5 × buffer, 1.25 mM MgCl2, 250 μM of each dNTP, 37.5 pmol of each primer (CYTb1 and CYTb2), and 0.5 U Taq DNA Polymerase (Invitrogen). Cycling conditions for the second round were as follows: 5 min at 95 °C; 30 sec at 94 °C; 30 sec at 45 °C; 1 min 30 sec at 72 °C (repeated for 35 cycles); 10 min at 72 °C. This combination of primers can amplify the cyt-b from other haemosporidian parasites infecting a diverse range of hosts (see Prugnolle et al., 2010, 2011; Boundenga et al., 2016; Makanga et al., 2016). All amplified products (10μl) were run on 1.5% agarose gels in Tris-acetate-EDTA (TAE) buffer. After analyze, the PCR-amplified products were used as templates for sequencing. DNA sequencing was performed Sanger method. All Plasmodium species identified in our study were mentioned in Table 1. Table 2 show the summary of the pairwise genetic distance estimation. This analyze was done to compare the divergence between sequence de cytochrome-b obtained in our study and sequences listed in Genbank.

To confirm host species, we performed molecular analyses to amplify cyt-b gene of rodents such as described in Refs. [5,6]. Thus, for amplification of cytb gene we used S330 (5′–CCAATGACATGAAAAATCATCG) and S331 (5′–GGGGATAGTCCTTCCTTCTTG). PCR conditions for an initial denature period of 94 °C for 2 min, followed by 35–40 cycles of 94 °C for 30–45 seconds, 55 °C for 45 seconds, and 72 °C for 1.5 minutes, and the reaction was terminated with a single cycle of 72 °C for 7 minutes. The phylogenetic tree was built with cyt-b sequences of rodent obtained and others rodent sequence known so far indexed in Genbank. All results are contained in Table 3, Table 4 S1 and Fig. 2.

## Appendix A Supplementary data

Multimedia component 1 Complete data base of captured rodents. It presents morphometric parameters, sex, ages and season of sampling. Multimedia component 1
